# Domestic violence and mental health: a cross-sectional survey of women seeking help from domestic violence support services

**DOI:** 10.3402/gha.v9.29890

**Published:** 2016-02-08

**Authors:** Giulia Ferrari, Roxane Agnew-Davies, Jayne Bailey, Louise Howard, Emma Howarth, Tim J. Peters, Lynnmarie Sardinha, Gene Solomon Feder

**Affiliations:** 1 Centre for Academic Primary Care, School of Social and Community Medicine, University of Bristol, Bristol, UK; 2 Domestic Violence Training Ltd, Surbiton, Surrey, UK; 3 Institute of Psychiatry, King's College London, London, UK; 4 School of Clinical Sciences, University of Bristol, Bristol, UK

**Keywords:** domestic violence and abuse, intimate partner violence, mental health, posttraumatic stress disorder, anxiety, CORE-OM, depression, women, advocacy

## Abstract

**Background:**

Domestic violence and abuse (DVA) are associated with increased risk of mental illness, but we know little about the mental health of female DVA survivors seeking support from domestic violence services.

**Objective:**

Our goal was to characterise the demography and mental health of women who access specialist DVA services in the United Kingdom and to investigate associations between severity of abuse and measures of mental health and health state utility, accounting for important confounders and moderators.

**Design:**

Baseline data on 260 women enrolled in a randomized controlled trial of a psychological intervention for DVA survivors were analysed. We report the prevalence of and associations between mental health status and severity of abuse at the time of recruitment. We used logistic and normal regression models for binary and continuous outcomes, respectively. The following mental health measures were used: Clinical Outcomes in Routine Evaluation – Outcome Measure (CORE-OM), Patient Health Questionnaire, Generalised Anxiety Disorder Assessment, and the Posttraumatic Diagnostic Scale to measure posttraumatic stress disorder (PTSD). The Composite Abuse Scale (CAS) measured abuse.

**Results:**

Exposure to DVA was high, with a mean CAS score of 56 (SD 34). The mean CORE-OM score was 18 (SD 8) with 76% above the clinical threshold (95% confidence interval: 70–81%). Depression and anxiety levels were high, with means close to clinical thresholds, and more than three-quarters of respondents recorded PTSD scores above the clinical threshold. Symptoms of mental illness increased stepwise with increasing severity of DVA.

**Conclusions:**

Women DVA survivors who seek support from DVA services have recently experienced high levels of abuse, depression, anxiety, and especially PTSD. Clinicians need to be aware that patients presenting with mental health conditions or symptoms of depression or anxiety may be experiencing or have experienced DVA. The high psychological morbidity in this population means that trauma-informed psychological support is needed for survivors who seek support from DVA services.

## Introduction

The reported lifetime prevalence of physical or sexual intimate partner violence (IPV), or both, for ever-partnered women varies globally from 15 to 71%, and the 12-month prevalence rates vary from 4 to 54% (1). One in five women aged 15 years or older has ever experienced IPV in Europe; 4% have experienced it in the past year (2). IPV is associated with depression, anxiety, posttraumatic stress disorder (PTSD), and substance abuse in the general population (3–5) and among women consulting in primary care (6, 7). There is evidence for a bidirectional effect (i.e. that women experiencing abuse are at greater risk of mental health conditions and that having a mental health condition makes one more vulnerable to abuse) particularly for depression, although there is a shortage of longitudinal studies to partition the directions of this effect (3). Qualitative research with survivors of IPV highlights the impact of abuse on the development of mental health problems (8). The few studies that have investigated the association between severity of exposure to IPV with mental and physical health problems reported positive associations (9–11). In these studies, the strength of association differed by type of abuse (9–12). Furthermore, Hegarty et al. (9) found that severe abuse is consistently associated with worse social coping, as well as increased levels of anxiety and posttraumatic stress symptoms. Abuse is also associated with poor self-reported physical health and pain, injuries, gynaecological and obstetric conditions, and difficulties carrying out daily activities (5, 13). Severity and type of PTSD (14) are also predicted by exposure to childhood abuse or maternal IPV (15).

Moreover, women who have recently experienced severe episodes of violence generally experience high levels of distress (9). Female survivors of IPV who seek advocacy support report high levels of abuse and depression when they first contact services (16, 17), higher than the general population (18). These levels decrease in time, independently of whether women are offered treatment or not (19, 20), and depression rates in women who have left a violent relationship up to 1 year earlier are similar to those in the general population (4).

Age may be a confounding factor in the relationship between exposure to IPV and mental health. Although younger women are at greatest risk of current abuse, older women have a greater lifetime experience; both current and lifetime experience increase the risk of mental health problems. Higher education and employment status are probably protective factors against IPV exposure (21–23). Socio-economic status, as well as recency and duration of abuse, therefore needs to be included in any analysis of the relationship between IPV exposure and mental health.

In this study, we aim to 1) characterise the demography and mental health of women who access specialist domestic violence and abuse (DVA) services in England and Wales; 2) investigate associations between the severity of abuse and measures of mental and physical health and quality of life, taking into account important potential confounders such as age and socioeconomic status, as well as important potential moderators such as exposure to direct maltreatment as a child (7, 21, 24) and prior history of mental health problems (3, 4).

## Methods

### Study setting and design

This study uses data from a cross-section of 260 women seeking help from two DVA services in the voluntary (non-statutory) sector in two UK cities, Bristol and Cardiff. Study participants were women recruited to the PATH (psychological advocacy towards healing) randomised controlled trial, testing the effectiveness and cost-effectiveness of a novel psychological intervention for survivors of DVA. Treatment was delivered by advocates or support workers called *specialist psychological advocates* in view of the specialisation they gained through the PATH training. Here we present findings from the baseline data we collected at recruitment. Sample size was determined by the need to detect reliable change in the main outcomes of the PATH trial (25). In this paper, the precision of the analysis is indicated by the confidence intervals of the estimated prevalence and associations.

Eligible participants were women 16 years or older who were experiencing DVA, which led them to seek support from a DVA agency in Bristol or Cardiff between 11 April 2011 and 4 June 2013. This included women who had experienced IPV or abuse (psychological, physical, sexual, or financial) from adult family members. Their first point of contact with the agencies, a support worker, screened them for other exclusion criteria: 1) psychotic illness; 2) severe drug or alcohol problem; 3) inability to read English; 4) current counselling, cognitive behavioural therapy, or other psychological treatments either in primary care or specialist psychiatric services.

Eligible women willing to discuss participation in the study were then contacted by a researcher who sought consent. At that meeting, women who consented to participation self-completed the baseline questionnaire on which this paper is based.

### Data collection

The PATH baseline questionnaire contained validated measures of mental health and exposure to abuse from an intimate partner, a member of the woman's family, or another adult. It also contained questions on socio-economic variables including age, parity, and employment status; substance use and general health variables; and measures of childhood exposure to abuse and maltreatment (24). A researcher was present in the room when the women filled in the questionnaire, to provide assistance if requested.

### Measurement

We used six scales to measure mental health (see Supplementary file). Symptoms of psychological distress were captured with the Clinical Outcomes in Routine Evaluation – Outcome Measure (CORE-OM), which measures symptoms of psychological distress in four domains: subjective well-being, problems and symptoms, functioning, and risk to self or others (26). CORE-OM is a standard screening measure in counselling services across the United Kingdom (26), and there are normative values from general and clinical populations in the United Kingdom. We used the continuous clinical CORE-OM score, with values between 0 and 40 (26).

We measured symptoms of depression with the nine-item version of the Patient Health Questionnaire (PHQ-9). The PHQ-9 is routinely used in general practice in the United Kingdom to screen for symptoms of depression, and there are normative values for both clinical and general populations (27). We computed an indicator equal to 1 if the PHQ-9 score was greater than 9, that is, suggestive of major depression (28). Symptoms of anxiety were measured with the seven-item Generalised Anxiety Disorder questionnaire (GAD-7) (29). We computed an indicator equal to 1 if the GAD-7 score was greater than 9. We measured posttraumatic stress with Foa's Posttraumatic Diagnostic Scale (30), and adopted the threshold recommended for this population (at least 17 points) for our analysis on the binary outcome (14). The EuroQol EQ-5D-5L (31) measured health state utility on a scale from less than 0 (worse than dead) to 1 (perfect health). Finally, we measured quality of life with the SF-12 (acute form), a measure of health status. Specifically, we computed the SF-12 aggregate mental and physical health sub-scales, which capture respondents’ physical and emotional health state and indicate whether these interfere with their daily lives and activities (32).

The measure of DVA was the Composite Abuse Scale (CAS). The CAS is a 30-item self-reported measure capturing emotional, physical, and severe abuse, as well as harassment (33). For our analysis we used a continuous version of the score, which can range between 0 and 150 (see Supplementary file). We preferred the continuous score to the binary (cut-off score: CAS ≥ 3) because of the high IPV exposure in our sample.

Recency of exposure was summarised by an ordinal variable that assigned higher values to more recent events. It varies between 0 (more than 12 months ago) and 4 (in the past month). Length of exposure varies between 1 (one occasion only) and 6 (for more than 5 years), increasing in the length of exposure. We summarised childhood abuse with a variable equal to 1 if the respondent had been the victim of either physical or sexual abuse in childhood. We also included a binary variable that denoted exposure to domestic abuse from a family member who was not an intimate partner, in order to account for exposure to multiple forms of abuse. Past mental health issues were self-reported by the women: the questionnaire asked whether they had experienced mental health problems such as depression or anxiety in the past. We coded all positive responses to this question as 1, and attributed a 0 score to all women who reported no problems. We used binary variables to capture whether the women had children younger than 4 years of age living with them and whether they were in a relationship. The indicator for cannabis use was set to 1 if the woman had used cannabis in the previous 12 months. We measured alcohol consumption with the AUDIT-C (Alcohol Use Disorders Identification Test – Consumption) instrument. We used a cut-off point of 3, which is thought to perform better for women and detects hazardous drinking (34). The women's age was measured in years, and their educational attainment with a categorical measure varying between 0 (no education) and 5 (bachelor's degree or higher). Their employment status was measured with a binary variable equal to 1 if the interviewee was not in work, that is, either unemployed, a student, or a retiree.

### Analysis

The data from the questionnaire were entered into an Access database. The CORE-OM and PHQ-9, together with the urban centre and type of service variables, were entered twice independently to ensure accuracy. Consistency and logical checks were performed in Access.

All analyses were conducted in Stata 12.1 (35). We characterised the sample with descriptive statistics of all variables.

For continuous variables, coefficients and 95% confidence intervals were calculated with normal regressions. For binary variables, odds ratios and 95% confidence intervals were calculated with logistic regressions. We report the univariable odds ratios (coefficients) with 95% confidence intervals for associations between mental health and exposure to abuse. The odds ratio (coefficient) and 95% confidence intervals of the adjusted estimates accounted for age, education, employment status, relationship status, the presence of children younger than 4 years of age, alcohol and drug use, and help-seeking for mental health in the past (36). We also adjusted for non-IPV domestic abuse and childhood abuse, as well as recency and duration of exposure. To investigate whether recency, duration, or child maltreatment modified the association between exposure and mental health, we also tested for multiplicative effects (data available upon request). All adjusted estimates also account for site (Bristol, Cardiff) and service type (refuge, outreach services) to reflect stratification in the sample (25). We present a complete case analysis, so that all women who had not reported a value for one of the variables in the model were excluded from the analysis. The number of respondents used to compute the statistics is always reported. We also excluded from analysis the seven women (out of 251) who reported experiencing DVA only from other family members and not from intimate partners.

### Ethical considerations

The study was approved by the South West National Research Ethics Service with specific approvals being received from appropriate local research ethics committees. Informed consent was sought from each woman during the first meeting, before she filled in the questionnaire, and the research assistant offered support in case of distress while the questionnaire was being completed.

## Results

The participating DVA services reported a total of 1,940 women requesting support during the recruitment period. We screened 66% of these women and 1,096 (86%) were eligible. Of these, 792 (72%) were approached and 263 (33%) recruited into the study. Three withdrew and 260 completed the baseline questionnaire, 13% of the women who originally requested support (Table 1). Language barriers and being in receipt of a psychological treatment accounted for 81% of ineligible cases (9% of initial throughput); time commitment represented the most common single reason why women declined recruitment after having been offered inclusion in the study.

**Table 1:** Recruitment

	Cardiff					Bristol				Total	
			
	Women's centre	Community outreach	Residential	Total	% of entered	Community outreach	Residential	Total	% of entered	*N*	% of entered
**Entered service**	**444**	**534**	**317**	**1295**		**519**	**126**	**645**		**1940**	
**Screened**	**162**	**408**	**209**	**779**	**60**	**372**	**121**	**493**	**76**	**1272**	**66**
*Ineligible*	*31*	*73*	*20*	*124*	*10*	*49*	*30*	*79*		*203*	*10*
Drugs and alcohol	6	9	1	16		7	0	7		23	1
Language barrier	14	14	7	35		20	27	47		82	4
Male	1	0	0	1		0	0	0		1	0
Psychotic	1	5	1	7		7	0	7		14	1
Psychological therapy	9	46	11	66		15	3	18		84	4
*Eligible but not approached*	*115*	*51*	*52*	*218*	*17*	*67*	*19*	*86*	*13*	*304*	*16*
One-off contact	29	0	0	29						29	1
SPA capacity	60	53	42	155		50	17	67		222	11
Researcher capacity	22	8	6	36		14	1	15		51	3
Other	4	19	4	27		3	1	4		31	2
**Unable to contact/declined**	**4**	**59**	**58**	**121**	**9**	**65**	**10**	**75**	**12**	**196**	**10**
**Approached**	**16**	**284**	**137**	**437**	**34**	**274**	**81**	**355**	**55**	**792**	**41**
*Did not consent to contact*	*6*	*117*	*15*	*138*	*11*	*115*	*26*	*141*	*22*	*279*	*14*
*Consented to contact*	*10*	*167*	*122*	*299*	*23*	*159*	*55*	*214*	*33*	*513*	*26*
**Met with researcher**	**6**	**108**	**64**	**178**	**14**	**92**	**45**	**137**	**21**	**315**	**16**
*Recruited*	*4*	*95*	*47*	*146*	*11*	*86*	*31*	*117*	*18*	*263*	*14*
*Not recruited*	*2*	*13*	*17*	*32*	*2*	*6*	*6*	*20*	*3*	*52*	*3*
Wanted counselling	1	2	3	6		0	*–*	0		6	0
Time commitment	1	6	5	12		2	2	4		16	1
Other	0	5	9	14		4	4	16		30	2
*Withdrawal*	*–*	*–*	*–*	*0*		*2*	*1*	*3*		*3*	*0*
**Total**	**4**	**95**	**47**	**146**	**11**	**84**	**30**	**114**	**18**	**260**	**13**

SPA, specialist psychological advocates.

For 26 of the 28 variables used in this analysis less than 10% of values are missing. The variable with the highest percentage of missing values is income (40%). In this paper we present the complete case analysis, and therefore we exclude income from the variables in our model, as we have two other measures of socio-economic status: level of education and employment. The women in our sample were 33 years old on average (Table 2); the majority had gained a City & Guilds diploma; almost 80% were not in formal employment.

**Table 2:** Sociodemographic profile of the sample

	Mean	Median	%	Minimum	Max	Standard deviation (IQR)	*N*
Age	33	31		18	63	17	248
Maximum education level		City & Guilds and similar		None	Bachelor's degree or higher	(GCSE to A-level)	233
Income bracket		Up to £10,999		Up to £10,999	More than £60,000	(Up to £10,999, to £11,000–£20,999)	156
White			87			34%	253
Currently in a relationship			20			40%	250
Perpetrator is current partner			23			42%	236
Is a parent			81			39%	254
Has children under 4 years of age			37			48%	260
Works in the household			38			49%	237
Not in formal employment (excluding retirees and students)			78			42%	236
Hazardous drinking (AUDIT-C ≥ 3)			54			50%	251
Smoked cannabis in past 12 months			26			44%	245
Witnessed DVA as a child			52			50%	257
Was abused as a child			50			50%	257
Had a mental health problem in the past			82			38%	251

IQR, interquartile range; DVA, domestic violence and abuse.

Almost 70% of the women reported severe abuse, with an overall average of 57 on the continuous CAS measure (Table 3). Abuse episodes were relatively recent and had been sustained over time for the majority of women. Out of 251 women, 7 reported being victims of domestic abuse from another member of the family and not from an intimate partner (2.8%; 95% confidence interval: 1.0%, 4.5%).

**Table 3:** Exposure to abuse

	CAS measure							
	
	Mean	Median	%	SD	Minimum	Maximum	Interquartile range	*N*
Severe abuse	6	3		8	0	33		248
Emotional abuse	31	31		16	0	55		248
Physical abuse	13	11		10	0	35		248
Harassment	8	7		6	0	20		247
Total abuse	57	49		34	0	136		245
Severe abuse >1			69	46%				248
Emotional abuse >3			96	20%				248
Physical abuse >1			92	28%				248
Harassment >2			86	35%				247
Total abuse >3			97	18%				245
Type of abuse, ordinal measure		SCA			None	SCA	(Physical and other – SCA)	251
Recency		In the past 3 months			More than 1 year ago	Past month	Between 6 months and less than 1 month ago	243
Length of exposure		Up to 3 years			Never	More than 5 years	Between (up to) 1 to more than 5 years	244

CAS, Composite Abuse Scale; SCA, severe combined abuse.

Two-thirds of the women reported clinical levels of psychological distress, with the total CORE-OM averaging 18 points (standard deviation: 7). At least 40% of women reported clinical levels of distress in all subareas of the CORE-OM, and at least 70% reported depression or anxiety symptoms (Table 4). Of 256 women, 197 (77%; 95% confidence interval: 71.2 to 82.9%) scored at least 17 points on the PTSD measure, the optimal threshold to identify this disorder (14), and 211 out of 256 (82%, 95% confidence interval: 77.6 to 87.1%) scored at least 15 points, the cut-off point recommended by Sheeran and Zimmerman (2002, in (14)). The measure of health state utility records a value of 0.6 (standard deviation: 0.3). Women in the general UK population have average EQ-5D values between 0.81 and 0.94 in the age groups below 64, and never lower than 0.71 in older women (37). Finally, quality of life measures suggest somewhat worse mental and physical health states compared to the general US population (32).

**Table 4:** Mental health, health utility, and quality of life measures

	Mean	SD	Median	Minimum	Maximum	*N*
CORE-OM						
Subjective well-being	24	8	25	3	40	
Percentage with mean ≥1.77		74%				259
Problems	22	10	23	0	40	
Percentage with mean ≥1.62		70%				259
Functioning	20	8	20	2	36	
Percentage with mean ≥1.3		80%				259
Risk	4	7	0	0	30	
Percentage with mean ≥0.31		41%				259
CORE-OM	18	7	19	2	35	
Percentage with mean ≥1.29		76%				259
Depression, anxiety, stress						
Depression (PHQ-9)	14	7	14	0	27	
PHQ-9 score >9		72%				258
Anxiety (GAD-7)	13	6	14	0	21	
GAD-7 score >9		70%				255
Post-traumatic stress (PTSD test for civilians)	26	12	27	0	50	
PTSD score ≥17		77%				256
Utility						
EQ-5D-5L	0.6	0.3	0.7	−0.2	1.0	249
Quality of life						
SF-12 Aggregate physical health	48	12	51	19	68	236
SF-12 Aggregate mental health	31	14	30	6	62	236

CORE-OM, Clinical Outcomes in Routine Evaluation – Outcome Measure; PHQ-9, nine-item Patient Health Questionnaire; GAD-7, seven-item Generalised Anxiety Disorder questionnaire; PTSD, posttraumatic stress disorder.

The crude associations of severity of exposure to abuse with mental health distress and trauma are strong (correlation coefficient: 0.3 and 0.4 respectively, *p*<0.0001 in both cases), as is that with health state utility (−0.3, *p*<0.0001). Women who reported symptoms of depression reported an average abuse score of 61 (standard deviation: 33), compared to an average of 43 (standard deviation: 30) for women who did not report depression symptoms. Similarly, women who reported symptoms of anxiety recorded an average exposure score of 61 (standard deviation: 34), compared to an average of 46 (standard deviation: 30) for women with no reported symptoms of anxiety. The remainder of this section reports results from linear and logistic regressions of mental health states on exposure to abuse, controlling for modifiers and sociodemographic characteristics.

Table 5 shows positive associations between exposure to abuse and psychological distress and negative associations between health state utility and quality of life and abuse, all measured with good levels of precision, except for the mental health subcomponent of the SF-12 and the measure of depression, once we adjusted for confounders.

**Table 5:** Associations between mental health and health state utility and severity of exposure to violence

Variable	Coefficient	Adjusted coefficient
Measures of mental health		
CORE-OM	0.081	0.1
95% CI	(0.050, 0.10)	(0.043, 0.2)
p value	0.004	0.013
*N*	245	174
PTSD	0.2	0.2
95% CI	(0.1, 0.2)	(0.1, 0.2)
p value	0.004	0.002
*N*	243	172
Measures of health state utility		
EQ-5D	−0.0028	−0.0037
95% CI	(−0.0038, −0.0018)	(−0.0052, −0.0023)
p value	0.003	0.003
*N*	238	170
Quality of life		
Aggregate physical health (T score)	−0.080	−0.093
95% CI	(−0.12, −0.040)	(−0.17, −0.012)
p value	0.008	0.035
*N*	228	165
Aggregate mental health (T score)	−0.10	−0.12
95% CI	(−0.18, −0.026)	(−0.23, 0.015)
p value	0.023	0.036
*N*	228	165

The first column of results reports coefficients from a normal univariable regression of the mental health or utility variable (COREOM, PTSD, SF-6D, EQ-5D) on exposure to abuse as captured by a continuous measure of the Composite Abuse Scale (CAS); the second column reports coefficients from a regression of CORE-OM, EQ-5D, SF-6D, and PTSD on CAS, and sociodemographic confounders (age, number of live-in children under 4, maximum level of education, use of drugs and alcohol, and work status) as well as measures of recency and length of exposure, previous mental health issues, exposure to non-IPV domestic abuse, and exposure to child abuse.

The severity of psychological distress increased with the severity and extent of abuse: for every additional point in the abuse score, women reported an increase of 0.081 points in the psychological distress score (*p*=0.004). Controlling for moderators such as childhood abuse, which increases the likelihood of exposure to abuse in adulthood (38), and sociodemographic characteristics slightly increased the size of this association, only slightly reducing the precision of the estimate.

The unadjusted association between exposure to abuse and posttraumatic stress was positive, with the measure of PTSD increasing 0.2 of a point for every unit increase in the measure of exposure to abuse (*p*=0.004). The size of this association was unchanged when we controlled for moderators and demographic characteristics.

Both measures of health state utility decreased as severity to exposure increased, with good precision for the physical health subcomponent of the SF-12 (*p*=0.008); precision was reduced once sociodemographic confounders were accounted for.

Associations between increasing exposure to abuse and symptoms of anxiety were positive and precisely estimated (Table 6).

**Table 6:** Associations between binary mental health states and severity of exposure to violence

Variable	Odds ratios	Adjusted odds ratios
PHQ-9 ≥ 10	1.02	1.03
95% CI	(1.01, 1.03)	(0.99, 1.05)
p value	0.002	0.113
*N*	244	174
GAD-7 ≥ 10	1.02	1.03
95% CI	(1.01, 1.02)	(1.01, 1.05)
p value	<0.0001	<0.0001
*N*	241	174
PTSD ≥ 17	1.03	1.03
95% CI	(1.02, 1.03)	(1.03, 1.04)
p value	<0.0001	<0.0001
*N*	243	172

The first column of results reports odds ratios from a univariable logistic regression of the mental health variable (PHQ-9, GAD-7, PTSD) on exposure to abuse as captured by a continuous measure of the Composite Abuse Scale (CAS); the second column reports adjusted odds ratios from a logistic regression of PHQ-9, GAD-7, and PTSD on CAS, and sociodemographic confounders (age, number of live-in children under 4, maximum level of education, use of drugs and alcohol, and work status) as well as measures of recency and length of exposure, previous mental health issues, exposure to non-IPV domestic abuse, and exposure to child abuse.

Unadjusted odds ratios suggest a small positive association between exposure to abuse and depression (odds ratio 1.02; 95% confidence interval 1.01 to 1.03). Adjusting for confounders leaves the association unchanged, but reduces the precision of the estimate.

The association with anxiety and PTSD is more precisely estimated than the one with depression. The univariable associations between exposure and the measures of anxiety and posttraumatic stress are positive. Controlling for moderators and other socio-economic variables suggests that the odds of being anxious or suffering from posttraumatic stress increase by 3% for every additional point in the score of exposure to abuse (95% confidence intervals: 1.02 to 1.05 and 1.03 to 1.04, respectively).

In our analyses, none of the tests for interactions between severity of abuse and recency, length of exposure, and child maltreatment were statistically significant (data available from authors).

## Discussion

Half of the women in our sample of IPV survivors had been exposed to IPV for up to 3 years and had experienced the last episode in the 3 months prior to getting in touch with the services. Half had been abused as children and more than four in five had had a mental health problem in the past. More than three-quarters reported symptoms of PTSD at the time they filled in the questionnaire. This finding is consistent with Howard and colleagues’ systematic review of epidemiological studies of diagnosed mental illness that reported the risk of PTSD as higher among women exposed to IPV than any other mental health condition. This is an important finding for clinicians, particularly generalists, who often miss the symptoms of PTSD in the context of domestic violence (39). Given the ubiquity and severity of PTSD resulting from IPV (40), health services need to develop and implement specific IPV trauma interventions for survivors.

The participants in our study had substantially more psychological distress, as measured by the CORE-OM, than the general and clinical populations of women in the United Kingdom. Their average score was four times higher than women in the general population, whose mean value is 4.8, and similar to women seeking psychological therapies in primary and secondary care, whose mean is 18.6 (41). The proportion of women who presented symptoms of depression in our sample was twice as large as that of women in UK general practice (27); for symptoms of anxiety, this proportion was three times as large (29). This profile is consistent with previous findings on women who seek advocacy support in the United States (16, 17) and Hong Kong (18).

Also consistent with other studies, we found that increasing severity of IPV was associated with worse mental health (10, 11, 36), especially anxiety and PTSD, even after controlling for confounders. In our population, exposure to recent IPV has a stronger association with symptoms of mental health illness than other known predictors: exposure to child maltreatment (3, 21), heavy drinking (23), or drug abuse (42), as well as a history of poor mental health.

Presentation of symptoms of mental illness in generalist or psychiatric practice should be considered a potential indicator of past or current IPV, or possibly non-partner domestic violence. It should prompt questions about abuse, as recommended in the WHO guidelines on intimate partner and sexual violence: ‘[H]ealth-care providers should ask about exposure to intimate partner violence when assessing conditions that may be caused or complicated by intimate partner violence’ (43) including symptoms of depression, anxiety, PTSD, sleep disorders, suicidality, or self-harm.

We found a very small negative association between increasing exposure to DVA and our health-related utility measures. One explanation for this may be that this measure is not appropriate for capturing the health and quality-of-life-related impacts of exposure to DVA in a highly traumatised population. For example, some of the domain-specific items on the EQ-5D, such as ‘I have [slight/moderate/severe] problems washing or dressing myself’ are not likely to be relevant to this population.

The strengths of our study include its focus on women seeking help for DVA, providing a basis for designing interventions for that group; its relatively precise estimates of the association between DVA severity and symptoms of mental illness; and the relatively low proportion of missing data, with the exception of income, which we replaced with education level and employment status to include socio-economic status in the analysis. These two variables are positively associated with income in the general population.

A limitation of our study is that the women in our sample are a minority of the women who presented at the participating DVA services and may differ from the women who were not eligible for the trial, were not approached, or declined to participate. In terms of the main findings of our study – the high proportion of survivors of IPV with symptoms of mental illness and the association of these symptoms with severity of violence – it is likely that the potential bias is in a conservative direction: women receiving psychological therapy or with psychotic symptoms (5% of women expressing interest in participation) were excluded. However, as potential participants were being offered psychological therapy in the context of the trial, it is likely that women with more psychological distress would be more likely to consent. A more general limitation is that our findings cannot be extrapolated to the whole population of women who have experienced DVA, as only a minority seeks help from DVA services.

Overall, our findings are consistent with other studies on the association between IPV and mental health problems.

The high mental health morbidity among women seeking support from DVA services highlights the need for effective, trauma-informed support services for this population. Equipping non-specialist support workers in advocacy agencies with psychological skills to support survivors of IPV may represent an important avenue for improving survivors’ well-being (44). Furthermore, particularly in resource-poor settings, upskilling non-specialist and non-medical personnel to deliver psychosocial support to women survivors of DVA may help engage hard-to-reach populations in a sustainable service framework. Were such interventions effective, they would very likely be cost-effective at improving survivors’ well-being, given the high cost of IPV to individuals, health services, and society as a whole (45).

## Supplementary Material

Domestic violence and mental health: a cross-sectional survey of women seeking help from domestic violence support services
