# Supplementary material for: Domestic violence and mental health: a cross-sectional survey of women seeking help from domestic violence support services
Source: Glob Health Action. 2016 Feb 8;9:10.3402/gha.v9.29890. doi: 10.3402/gha.v9.29890 (PMC4748088; doi:10.3402/gha.v9.29890)
Supplement: Domestic violence and mental health: a cross-sectional survey of women seeking help from domestic violence support services [file GHA-9-29890-s001.pdf]

Supplementary file

Table of measures for web appendix

Measure	Measurement	Description
CORE-OM<br><br>Clinical Outcomes in Routine Evaluation—Outcome Measure<br><br>(1)	Continuous clinical measure:<br><br>In-sample range: (2, 35)	Measures symptoms of psychological distress. It is non-proprietary and was originally designed to measure the impact of counselling and psychological therapies on patients in the UK. It is made of 34 items, all measured on a Likert scale, with higher scores corresponding to worse states (some items are reversed). It captures four domains:<br><br>i. *subjective wellbeing:* measures respondent's emotional wellbeing (4 items)<br><br>ii. *problems/symptoms*: questions on anxiety, depression, physical issues and effects of trauma (12 items);<br><br>iii. *functioning*: captures respondents' perceptions of their close relationships, general emotional functioning, and their social interactions (12 items);<br><br>iv. *risk to self or others*: captures if respondents' behaviour may have caused harm to themselves or others (6 items)
PHQ-9<br><br>Nine—item Patient Health Questionnaire<br><br>(2)	Binary:<br><br>0 if PHQ--9 =< 9<br><br>1 if PHQ--9 >9	The PHQ--9 is used routinely in general practice in the UK to screen for symptoms of depression.<br><br>It includes questions on respondents' perceptions of their own energy levels, worrying patterns, and self-esteem, among others.<br><br>It measures how often respondents have felt the way described by each of the items. Each item is scored on a Likert scale, where higher scores indicate higher frequency, equivalent to worse health states.<br><br>We computed an indicator equal to 1 if the PHQ--9 score was greater than 9, i.e. suggestive of major depression.
GAD-7<br><br>Seven—item Generalised Anxiety	Binary:<br><br>0 if GAD-7 =< 9	The GAD—7 measures symptoms of anxiety. Each item is scored on a Likert scale, with higher scores indicative of higher frequency spent in the state indicated by the item, i.e. of worse mental health states. Items capture a sense of generalised fear, nervousness and worrying, among others.

Disorder questionnaire (GAD7)<br><br>(3)	1 if GAD-7>9	
CAS<br><br>Composite Abuse Scale<br><br>(4)	continuous<br><br>In-sample range: (0, 136)	The CAS is a 30--item self--report measure capturing emotional, physical and severe abuse, as well as harassment (4)(4)(32).<br><br>It was designed to measure exposure to coercive control and conflict among women, in response to critiques to existing measures that they only measured conflict and physical or sexual violence.<br><br>Items are measured on a Likert scale, where higher scores indicate more frequent exposure to the abuse described by the item, and hence higher exposure to abuse.<br><br>The cut-off score for CAS has been established at 3. Due to the high level of exposure to abuse in our sample, we use a continuous measure
PTSD Check List<br><br>Post--traumatic stress<br><br>(5) (6)	Continuous<br><br>In-sample range: (0, 50)	Weathers' measure of post--traumatic stress is a 12-item checklist that captures the presence of intrusive thoughts regarding respondents' traumatic experiences, feeling of severe anxiety or lack of emotions, of social isolation, and general pessimism or lack of hope for the future. Each item is measured on a Likert scale that captures how frequently respondents have experienced the state or feeling described by each item, with higher values indicative of higher frequencies, and hence of worse post—traumatic stress.
SF-12<br><br>Short-Form 12<br><br>(7)	Two sub-categories, continuous:<br><br>Aggregate Mental Health T-Score:<br><br>In-sample range: (6, 62)<br><br>Aggregate Physical Health T-Score:<br><br>In-sample range: (19,68)	The SF-12 is a measure of health status that captures respondents' physical and emotional state and whether these interfere with their daily lives and activities. All indicators are measured along Likert scales where higher scores are indicative of better health status.<br><br>Key dimensions are:<br><br>A. Most closely associated with the physical health dimension (Physical Component Summary Measure (PCS)):<br><br>i. *Physical Functioning*: measures the degree and type of difficulties respondents encounter when performing mild exertion (2 items)

		ii. *Role-Physical*: measures if physical health state interferes with individuals' performance of their role in society (work or usual activities) (2 items)<br><br>iii. *Bodily Pain*: measures how far physical pain hampers work (1 item)<br><br>iv. *General Health:* measures respondents' overall assessment of their health state (1 item)<br><br>B. Most closely associated with the mental health dimension (Mental Component Summary Measure (MCS)):<br><br>i. *Vitality*: rates respondents' perception of their own level of energy (1 item)<br><br>ii. *Social Functioning*: interference of physical or emotional health with respondent's social activities (1 item)<br><br>iii. *Role-Emotional*: measures the interference of emotional health with respondents' amount and quality of work or other activities (2 items)<br><br>iv. *Mental Health:* measures nervousness and depression (2 items)
EQ5D—5L<br><br>Five dimensions five levels EuroQol measure<br><br>(8)	Continuous<br><br>In-sample range: (-.218, 1),<br><br>where<br><br>-.218= 44452=worse health state<br><br>1=11111=perfect health	The EuroQol EQ5D—5L measures health state utility by assigning a utility value to each of the possible 3125 health states the measure describes. The health states are mapped over 5 dimensions:<br><br>i. mobility,<br><br>ii. self-care,<br><br>iii. usual activities,<br><br>iv. pain or discomfort, and<br><br>v. anxiety and depression.<br><br>Respondents rate how they fare along dimension on a 1-5 Likert scale, where 5 is worst and 1 is best.<br><br>The final continuous score is assigned on the basis of population scores derived from large surveys.

REFERENCES

1. Barkham M, Mellor-Clark J, Connell J, Cahill J. A core approach to practice-based evidence: A brief history of the origins and applications of the COREOM and CORE System. Counselling and Psychotherapy Research. 2006; 6(1):3-15.
2. Kroenke K, Spitzer RL. The PHQ-9: A New Depression Diagnostic and Severity Measure. Psychiatric Annals. 2002;32(9).
3. Spitzer RL, Kroenke K, Williams JBW, Loewe B. A Brief Measure for Assessing Generalized Anxiety Disorder, The GAD-7. Archives of Internal Medicine 2006;166:1092-7.
4. Hegarty K, Fracgp, Bush R, Sheehan M. The composite abuse scale: further development and assessment of reliability and validity of a multidimensional partner abuse measure in clinical settings. Violence Vict. 2005;20(5):529-47.
5. Weathers FW, Keane TM, Davidson JRT. Clinician -administered PTSD scale: A review of the first ten years of research. Depression and Anxiety. 2001;13(3):132-56.
6. Griffing S, Lewis CS, Chu M, Sage RE, Madry L, Primm BJ. Exposure to Interpersonal Violence as a Predictor of PTSD Symptomatology in Domestic Violence Survivors Journal of Interpersonal Violence 2006;21.
7. Maruish ME. User’s manual for the SF-12v2 Health Survey Lincoln, RI: QualityMetric Incorporated, 2012.
8. Rabin R, de Charro F. EQ-5D: a measure of health status from the EuroQol Group. Annals of Medicine, The Finnish Medical Society Duodecim. 2001;33:337-43.
